# Using Machine Learning to Train a Wearable Device for Measuring Students’ Cognitive Load during Problem-Solving Activities Based on Electrodermal Activity, Body Temperature, and Heart Rate: Development of a Cognitive Load Tracker for Both Personal and Classroom Use

**DOI:** 10.3390/s20174833

**Published:** 2020-08-27

**Authors:** William L. Romine, Noah L. Schroeder, Josephine Graft, Fan Yang, Reza Sadeghi, Mahdieh Zabihimayvan, Dipesh Kadariya, Tanvi Banerjee

**Affiliations:** 1 Department of Biological Sciences, Wright State University, Dayton, OH 45435, USA; graft.2@wright.edu; 2 Department of Leadership Studies in Education and Organizations, Wright State University, Dayton, OH 45435, USA; noah.schroeder@wright.edu; 3 Department of Computer Science and Engineering, Wright State University, Dayton, OH 45435, USA; yang.57@wright.edu (F.Y.); dipesh@knoesis.org (D.K.); tanvi@knoesis.org (T.B.); 4 Department of Electrical & Computer Engineering and Computer Science, University of New Haven, West Haven, CT 06516, USA; rsadeghi@newhaven.edu; 5 Department of Computer Science, Central Connecticut State University, New Britain, CT 06050, USA; zabihimayvan@ccsu.edu

**Keywords:** cognitive load, machine learning, wearable sensor, studying, learning analytics

## Abstract

Automated tracking of physical fitness has sparked a health revolution by allowing individuals to track their own physical activity and health in real time. This concept is beginning to be applied to tracking of cognitive load. It is well known that activity in the brain can be measured through changes in the body’s physiology, but current real-time measures tend to be unimodal and invasive. We therefore propose the concept of a wearable educational fitness (EduFit) tracker. We use machine learning with physiological data to understand how to develop a wearable device that tracks cognitive load accurately in real time. In an initial study, we found that body temperature, skin conductance, and heart rate were able to distinguish between (i) a problem solving activity (high cognitive load), (ii) a leisure activity (moderate cognitive load), and (iii) daydreaming (low cognitive load) with high accuracy in the test dataset. In a second study, we found that these physiological features can be used to predict accurately user-reported mental focus in the test dataset, even when relatively small numbers of training data were used. We explain how these findings inform the development and implementation of a wearable device for temporal tracking and logging a user’s learning activities and cognitive load.

## 1. Introduction

Monitoring engagement with learning materials in real time can be challenging. Many of us can likely recall instances where we had to re-read a paragraph because we were thinking about something else and had no recollection of what the paragraph said. We envision a system that helps learners identify when they are not putting appropriate mental effort into learning, and believe that real-time classification of one’s cognitive load could improve study habits. We describe the development and implementation of the Educational Fitness (EduFit) system, which utilizes physiological data from a wearable sensor and machine learning to differentiate between different levels of cognitive load for the user.

While the literature shows an abundance of research focused on applying the concept of cognitive load, mental effort, or mental workload to different domains, the measurement of cognitive load has remained rife with limitations throughout the educational literature [1,2]. Current popular measurement techniques are limited by subjectivity and can interfere with regular activities [3], or require the use of expensive, specialized equipment [4]. Recently, the use of basic physiological measures in learning contexts has drawn renewed attention by researchers, perhaps in part due to the growing availability of minimally invasive wearable sensors. For example, a decrease in heart rate between the beginning and end of a 60-min class may suggest that students lose alertness and neurocognitive excitability [5]. Further, electrodermal activity (EDA) has demonstrated usefulness in distinguishing between engaged and non-engaged students [6]. Recently, researchers used a multimodal approach, measuring EDA, heart rate, body temperature, and blood volume pressure to capture a student’s reported learning experience, and then examined these data in relation to satisfaction, perceived usefulness of the learning experience, and performance measures [7]. However, present studies do not explore the usefulness of physiological data for real-time classification of activity or reported cognitive load, which would be necessary for a wearable sensor that is useful for tracking cognitive load. We therefore describe the development, implementation, and validation of a non-invasive wearable multi-modal physiological sensor for real-time tracking and logging of cognitive load during learning tasks. In this article, we describe two studies. In the first study, we explore the efficacy of electrodermal activity, skin temperature, and heart rate for classifying three activities, which are associated with varying levels of cognitive load. In the second study, we evaluate the efficacy of these physiological measures for classification of whether or not a learner is focused on a task.

## 2. Review of Literature

### 2.1. Theoretical Framework

It is widely accepted that humans engage their working memory to evaluate new information [8,9]. This puts a cognitive burden on the working memory, a construct commonly known as cognitive load in the education literature, and working memory load or mental workload in other fields [10,11]. The overall premise of cognitive load theory is that the working memory only has a finite and limited number of resources available at any point in time, and they should ideally be used for processing the necessary information rather than distractions [8,9]. For the purposes of this paper, we refer to cognitive load as the amount of mental effort being expended on the task.

According to cognitive load theorists, mental effort can be expended in different ways when learning. Specifically, effort directed directly towards the learning task due to the complexity of the learning materials is known as intrinsic cognitive load, of which germane cognitive load is a subtype focused on the construction of mental models, whereas the mental effort directed towards learning that is due to the way the materials are presented is known as extraneous cognitive load [8,12]. The goal of instruction therefore is to minimize the extraneous load and moderate the intrinsic load to be at an appropriate level for the learner so one does not exceed their working memory capacity when completing the learning task [8,9,12].

### 2.2. Measuring Cognitive Load and Mental Workload

While cognitive load theory is conceptually relatable for many who design instruction, it is limited by theoretical and practical measurement issues. Self-report and secondary task measures have been the most commonly used methods. However, self-reports force the learner to stop the learning process and respond to a survey instrument [3]. Similarly, secondary task techniques have the potential to interfere with the primary learning task [13], meaning that they may have to be changed depending on how instruction is being provided [14], and they may be challenging to monitor depending on the task. Real-time physiological measures, including electroencephalography (EEG) [15], eye-tracking [16], and heart rate-related measures [4], avoid both of these problems but have often been viewed as invasive and lacking sensitivity [4].

Differentiating between the different types of cognitive load has also been a persistent challenge, and some have suggested that it may not be possible to distinguish between different load types [12]. As such, measuring overall cognitive load may be a more fruitful approach, and that is what some physiological measures are viewed to measure [15,17]. Accordingly, in this study we view the physiological data we collect as an indicator of overall cognitive load and do not claim to be measuring specific types (e.g., intrinsic or extraneous load).

As noted, cognitive load and mental workload are similar constructs. Although physiological measurement in learning or studying contexts is relatively new, it has been applied more widely in other fields. Since the autonomic nervous systems automatically and unconsciously respond to mental workload demands [18], physiological measures can be used as an index of workload [11]. While a variety of measures have been used in the literature, we focus on skin temperature, heart rate, and EDA in terms of galvanic skin response (GSR).

Skin temperature has been used as a non-intrusive measure of mental workload, although its use in the literature is sparse. For example, [19] examined how facial skin temperature changed during simulated and actual driving tasks, finding significant correlations between nose temperature and a subjective workload measure. Meanwhile, in [20], heart rate predicted changes in cognitive load correctly 62.5% of the time. In regards to GSR, [20] found that GSR could be used to classify mental effort with 75% accuracy. Finally, [3] examined the use of GSR in relation to educationally relevant tasks in math and reading. Their results showed that once the data were normalized, GSR could discriminate between tasks of different difficulties. However, despite these successes, GSR results have not always shown such promise [21].

As shown, a number of different physiological measures have been used to examine mental workload in fields other than education (for reviews, see [22,23,24]). However, a recent systematic review concluded that there was no one particular measure that may work well in all circumstances [24]. While multimodal measures have been used to predict cognitive load, few, if any, studies have extended the predictive function to an actual learning task. As the ubiquity of wearable fitness sensors continues to increase, it makes sense to explore the efficacy of these types of data for classification of cognitive load in education contexts.

### 2.3. Using Physiological Data in Education Contexts

The use of physiological data in education contexts is relatively limited, especially when compared to self-report approaches to measuring cognitive load, but some promising research does exist. For example, researchers have shown that heart rate and EDA are promising features for classification of mental engagement [5,6]. Specifically, in a study with 30 students using an Arduino-based sensor, heart rate was shown to provide a measure of alertness and neurocognitive excitability [5]. Meanwhile, Empatica E4 wearable sensor data from 24 students, 9 teachers, and 41 lectures showed that EDA can provide a useful measure of emotional arousal, which is associated with engagement or non-engagement in classroom contexts [6]. In addition, [25] examined EDA and skin temperature in relation to high and low complexity tasks. Using wrist-worn wearables and survey methods, they found that mean physiological scores did not differ by the complexity of the activity; however, EDA was positively correlated with self-reported mental effort when individuals completed the high complexity task. Yet, the low complexity task did not show the same significant correlation [25].

While these individual physiological measures show interesting results, only recently have multimodal physiological measurements been applied in educational research. As such, relatively few studies exist, but some have shown promising findings. For example, in a study of 31 students from 93 class sessions, [7] equated measures of heart rate, EDA, body temperature, and blood volume pressure collected using the Empatica E4 to self-reported 7-point Likert measures of perceived satisfaction, usefulness, and performance. Using temporal features within a support vector machine with a polynomial kernel, predictions within one Likert scale point could be obtained using an average of 25 min of data [7]. However, this study did not evaluate the data in real-time and did not focus on cognitive load.

### 2.4. The Present Studies

As discussed, physiological data has been used extensively for measuring cognitive load outside of educational contexts, yet the use of physiological data in education contexts has often been viewed as problematic for a multitude of reasons. Researchers have recently begun exploring the relations between physiological measures from wearable sensors and the learning process, but this work is limited. Furthermore, we are unaware of any studies that have used multimodal physiological data to predict cognitive load in real time, as would be required to create the cognitive load tracker we envision.

In this paper, we describe two studies that work towards a non-invasive, wearable sensor that can help a learner self-regulate their learning process. The first study aimed to examine if EDA, skin temperature, and heart rate are useful for the classification of activities hypothesized to represent different levels of cognitive load. This work is needed to help validate the use of these measures for multimodal cognitive load measurement. Building on these findings, the second study examined the efficacy of these measures for the classification of a learner’s self-reported focus on a task. Together, these studies provide a foundation for a wearable sensor that can monitor cognitive load using physiological data that is easily obtainable from wearable fitness trackers.

## 3. Study 1

Since few studies have examined the use of wearable sensors for the monitoring of cognitive load in learning contexts, and few studies have examined the use of different types of physiological data in this context, it is important to first confirm the extent to which these measures are useful for classifying activities associated with different levels of cognitive load. Above we discussed how few studies have utilized multimodal measures in this regard. As such, in Study 1 we sought to examine the extent to which three commonly used physiological measures, specifically Skin Temperature (TEMP), EDA, and Heart Rate (HR), could be used together to classify activities representing varying levels of cognitive load.

### 3.1. Methods

#### 3.1.1. Measurement of Physiological Parameters

We collected participant data with the Empatica E4, an unobtrusive wrist-worn physiological sensor. The E4 is similar to a traditional fitness tracker in design [26] and measures Blood Volume Pulse (BVP), Heart Rate (HR), Interbeat Interval (IBI), Skin Temperature (TEMP), 3-Axis Acceleration (ACC), and Electrodermal Activity (EDA). We focus on TEMP, EDA, and HR. EDA is the measurement of electrical conductance of the skin, also called galvanic skin response (GSR), for detection of autonomic nervous system arousal [27]. For this study, we used the amplitude of the participant’s EDA signal, which was output directly from the E4 device. The device measured EDA and TEMP at 4 Hz. HR (1 Hz) was calculated based on BVP (64 Hz). Since the respective activities, completed on the order of minutes, served as the ground truth labels, we downsampled EDA and TEMP to 1 Hz in order to match the HR sampling frequency. Although there was some loss of information in this downsampling process, we found this preferable to upsampling, which would add more noise to the data.

#### 3.1.2. Transformation of Physiological Features

Before using statistical models and machine learning, we put all features onto the same scale similar to [3]. With respect to our study design, this normalization process also needed to account for individual baseline and variability differences in TEMP, EDA, and HR. We therefore used z-standardization, which maps the features on a scale of standard deviations centered at the mean. In order to account for individual baseline and variability, we mapped each participant’s features on a scale of his/her standard deviation centered at his/her own mean across all trials. This ensured that each participant’s values for TEMP, EDA, and HR accounted for his/her unique variability with respect to his/her unique average value.

#### 3.1.3. Experimental Design

In Study 1, we were interested in the efficacy of TEMP, EDA, and HR in classifying a participant’s activity. We used three activities comprising different hypothesized levels of cognitive load (Table 1): (1) deep cognition—a state associated with active problem solving like completing a math problem or solving a puzzle; (2) leisured cognition—a state associated with passive cognitive activity like reading a novel or browsing the internet; and (3) daydreaming—a state associated with lack of focus on a particular task. To represent these different cognitive states, we asked participants to engage in three activities (Table 1): (1) playing Sudoku without distractions, which represents an activity with high cognitive load; (2) browsing the internet or checking e-mail, which represents an activity with moderate cognitive load, and (3) sitting and doing nothing, which represents an activity with low cognitive load.

Seven adults (4 male and 3 female), ranging from 27–38 years of age, participated in the study (descriptive statistics in Table 2). Three of the participants ranged from 26–28 years of age, two of the participants were 32 years of age, and one participant was 38 years of age. Each participant placed the E4 band on the wrist approximately 3 cm below the base of the hand. After approximately 2 min of wearing the E4, each participant was first asked to play a game of Sudoku at the difficulty level of their choice. Immediately after finishing the Sudoku game, they were asked to spend 15–20 min checking e-mail or browsing social media. After finishing this, they were asked to spend 10–15 min sitting and doing nothing, with no external forms of engagement. Two of the participants did these three activities twice, one participant did them 4 times, two participants did them 5 times, and two participants did them 6 times. This resulted in a total of 30 trials for each activity across the 7 participants. On average, each Sudoku session took 18 min and 16 s, each computer browsing session took 14 min and 41 s, and each period of non-engagement lasted 13 min and 18 s. Across all subjects, trials, and activities, this totaled 23 h and 7 min and 19 s of sensor data.

#### 3.1.4. Training and Testing of Classifiers

In order to understand how machine learning may be used in a physiological sensor designed for detecting the type of activity, we utilized a variety of machine learning algorithms, which can be distinguished into 3 types: (1) a constant model which uses the bias in the data as a predictor, (2) baseline models, which are designed to be conceptually simple and relatively easy to interpret, and (3) black box models, which contain many parameters and are comparatively difficult to interpret but can build more complex models and tend to outperform the baseline models.

The constant model, the simplest possible model, which makes predictions based on the most commonly occurring outcome, provided a lower bound for classification performance. Baseline models included logistic regression (weak L1 regularization: C = 1000), naïve Bayes, k nearest neighbors (Euclidean distance with k = 3), and decision tree (maximum depth = 4, no splitting of subsets smaller than 5, minimum number of instances in leaves = 1). Black box models included multi-layer neural network (sigmoidal activation function, 1 hidden layer with 100 neurons), support vector machine (SVM) (sigmoidal kernel, cost = 1, epsilon = 0.01), AdaBoost (10 estimators, learning rate = 1), and random forest (100 trees, no splitting of subsets smaller than 5). We used the scikit-learn toolbox in Python to fit the models. In the interest of evaluating the robustness of the models with new data, we used a shuffle-split process for validation, where 100 random samples were drawn with replacement from the data. To obtain a liberal estimate of performance, we first drew 95% of the data within each random draw to train the models, and then tested the models on the remaining 5% within each random draw. To obtain a more conservative estimate, we drew 10% of the data within each random draw to train the models, and then tested them on the remaining 90% of the data within each random draw. Strength of classification of the models on the test dataset was assessed through the average area under the receiver operator characteristic (ROC) curve (AUC), precision, recall, and F1 score, which is the harmonic mean of precision and recall [28], across the 100 random draws.

### 3.2. Results

#### Cognitive Task Prediction

Data from this study show that TEMP, EDA, and HR are useful for classifying a participant’s cognitive task under both conservative and liberal assumptions related to the number of training data available (Table 3).

Of the baseline models, k-nearest neighbors (KNN) was the best performing (AUC = 0.89–0.97, F1 = 0.84–0.93). The classification performance of the other three baseline models was moderate to poor, with AUC values below 0.7 and F1 values below 0.5. Of the black box models, random forest was the best performing (AUC = 0.93–0.99, F1 = 0.85–0.94), followed by AdaBoost (AUC = 0.78–0.93, F1 = 0.80–0.92).

### 3.3. Discussion

We were surprised that the SVM and neural networks performed relatively poorly given their capacity to model complex relationships between input features and the output classes, with classification performance paralleling that obtained from the baseline models. This potentially demonstrates the necessity of ensemble approaches such as AdaBoost and random forest for classification of learning activities within this type of framework. Random forest performed quite well even when very little training data were used (AUC = 0.93, F1 = 0.85), which demonstrates its utility for providing useful labels even when relatively small numbers of data are available. This is significant because many factors affect physiology, and it is often difficult to control a student’s environmental circumstances within a learning or studying situation. All of these aspects add noise to the data, and ensemble methods, such as AdaBoost and random forest tend to be robust to these types of noise by building several weak rules that can factor in the different conditions under which a certain activity or cognitive workload can occur. This is also a good place to point out that there is noise due to imperfect control of the experiment itself, which mirrors what the training process for the EduFit device for activity classification may entail. Namely since cognitive load was not reported directly by participants, there is likely individual variation in the levels of mental effort put forth in the respective activities. This seems particularly likely given that cognitive load can vary by individual due to their prior knowledge or familiarity with a task [8]. We did not set specific constraints on what the person was doing when browsing the internet, what she was thinking about when sitting and resting, or the difficulty of the Sudoku puzzle. The differing performance of these classifiers may reflect the limitation that we were not able to control the types of mental activities in which the participants were engaging, and corresponding cognitive load, as they were sitting at rest or browsing the internet. An experiment that utilizes self-report measures of cognitive load in response to specific tasks may help overcome this limitation and improve model classification performance.

## 4. Study 2

The results from Study 1 showed that the amplitude of a person’s electrodermal activity, body temperature, and heart rate could be used to effectively classify activities representing varying levels of cognitive load. However, a limitation of this work was that there was not a self-reported measure from the participants for comparison. To address this limitation, in the second study, we were interested in the efficacy of TEMP, EDA, and HR for predicting self-reported mental focus in real time.

### 4.1. Methods

#### 4.1.1. Experimental Design

We used the Empatica E4 for data collection, and measurement and transformation of the physiological features was similar to Study 1. Given the efficacy of TEMP, EDA, and HR for predicting a participant’s activity in Study 1, the purpose of the experimental design in Study 2 was to obtain a self-report measure of mental focus from each participant in the context of a pre-defined set of activities (descriptive statistics in Table 4). Each participant completed similar activities while wearing the E4, and were asked to rate their level of mental focus after each activity. Study 2 involved 7 adult participants—5 female and 2 male. Four of the participants were 18–19 years of age, and one participant was 23 years of age. The other two participants were 39 and 49 years of age, respectively.

Using Qualtrics survey software, each participant was asked to complete five puzzles developed by the American Alzheimer’s Association, which the participants reported ranged in difficulty from extremely easy to extremely difficult. The five puzzles took participants an average of 10.6 (SD = 4.1) minutes to complete. Completion times ranged from 6.1 to 16.6 min. A total of 85 min of data were collected, which comprised a single trial for six of the participants, and two trials for one participant.

Before beginning the puzzles, each participant was asked to wear the Empatica E4 device for approximately 2 min. We placed the E4 band on the wrist approximately 3 cm below the base of the hand. After the participant completed each puzzle, she was asked to rate the level of mental focus given to the puzzle (not focused, somewhat focused, or focused). Since our interest is in a device that can detect a state of high cognitive load, we coded a report of high focus as ‘1′ and moderate focus or lack of focus as ‘0′ (reported in Table 4). Two of the participants (Table 4) indicated that they were focused the entire duration of the experiment. Although this lack of variance in the outcome would contraindicate successful training of a device on that single participant, we elected to retain these data since our training and testing procedure was not stratified at the participant level. These participants followed the experimental procedures, and we were interested in using as many data as possible in the classification models.

#### 4.1.2. Training and Testing of Classifiers

We utilized the bias in the data, four baseline models, and four black box models, to classify each participant’s self-reported mental focus (0 = not focused; 1 = focused).

Baseline models included logistic regression (weak L1 regularization: C = 1000), naïve Bayes, k nearest neighbors (KNN) (Euclidean distance with k = 3), and decision tree (maximum depth = 4, no splitting of subsets smaller than 5, minimum number of instances in leaves = 1). Black box models included multi-layer neural network (sigmoidal activation function, 1 hidden layer with 100 neurons), support vector machine (SVM) (sigmoidal kernel, cost = 1, epsilon = 0.01), AdaBoost (10 estimators, learning rate = 1), and random forest (100 trees, no splitting of subsets smaller than 5).

Since an individual’s actual level of cognitive load within a particular activity was not quantified in Study 1, the self-report measure for mental focus collected in Study 2 made way for an evaluation of how cognitive load affects a person’s TEMP, EDA, and HR. We began with studying the logistic regression model to understand the relationship between the physiological features (TEMP, EDA, and HR) and self-reported focus. This included the relative importance of each of the features as well as the direction of the relationships. We used binary logistic regression with a multi-level modeling approach to explore and control for individual cluster effects [29]. Using SPSS 21.0, we first fitted a main effects model that included TEMP, EDA, and HR as predictors for the outcome of focus. Next, we fitted an intercept-by-person model, which allowed each participant to have a unique model intercept while constraining the effects of TEMP, EDA, and HR to be similar across participants. Third, we fitted an intercept- and trend-by-person model, which allowed each participant to have both a unique intercept and unique trends. We evaluated the main effects using odds ratios with respect to the best fitting model (95% confidence). The odds ratio was defined as the effect of a one standard deviation increase in a predictor on the odds of reporting a high level of focus. Significance of the odds ratio was evaluated at the 95% confidence level.

We used the scikit-learn toolbox in Python to fit the machine learning models. As described previously for Study 1, in the interest of evaluating the robustness of the models with new data, we used a shuffle-split process for validation, where 100 random samples were drawn with replacement from the data. A liberal estimate of model performance was obtained by drawing 95% of the data within each random draw to train the models, and then testing the models on the remaining 5% within each random draw. To obtain a more conservative estimate, we drew 10% of the data within each random draw to train the models, and then tested them on the remaining 90% of the data within each random draw. The liberal approach represents a scenario where there are many data from each individual to train the classifier. The conservative approach represents a situation where there are relatively few data available, and where predictions for one individual may need to be made based on another individual’s data. Strength of classification was assessed through the area under the receiver operator characteristic (ROC) curve (AUC), precision, recall, and F1 score, which were averaged over the 100 random draws.

### 4.2. Results

#### 4.2.1. Statistical Modeling and Inference for Feature Importance

The logistic regression model containing only the main effects of EDA, HR, and TEMP fitted the data significantly better than simply using the outcome bias as a predictor (χ^2^ = 28.79, df = 3, *p* << 0.001). Allowing the outcome bias to change between participants (χ^2^ = 2527.09, df = 10, *p* << 0.001) significantly improved model fit over the model with just the main effects (difference χ^2^ = 2498.30, df = 7, *p* << 0.001). A model allowing both model bias and trends to change between participants (χ^2^ = 4224.09, df = 31, *p* << 0.001) improved model fit even further (difference χ^2^ = 1697.00, df = 24, *p* << 0.001). This may demonstrate the role that individual differences play in understanding how the body reacts to changes in cognitive load. It may also be demonstrative of differences in how individuals perceive their own state of mental focus, which may affect their self-reports.

When these individual differences were accounted for, the model indicated an increase in heart rate with increased mental focus (Table 5). A 1 standard deviation increase in an individual’s heart rate was found to increase the odds of being in a focused state 1.4 times (OR = 1.39). Conversely, body temperature and EDA were found to decrease with increased cognitive load. A 1 standard deviation increase in body temperature and EDA reduced the odds of reporting a focused state by 2.6 times (OR = 0.39) and 4.2 times (OR = 0.24), respectively. These values (Table 5) suggest that EDA and TEMP are relatively important predictors in comparison to HR when predicting mental focus on a task.

#### 4.2.2. Prediction of Self-Reported Mental Focus

When moving to the target variable of user-reported mental focus, we observe a similar trend as in Study 1 in terms of the classifiers which perform well, and those that struggle with classification (Table 6). Of the baseline models, KNN performs best (AUC = 0.81–0.87, F1 = 0.79–0.80). The similarity in classification performance under both conservative and liberal assumptions suggests that KNN may also perform well with limited data. Although it does not perform as well as KNN, the same can be said for the decision tree (depth = 4, AUC = 0.71–0.73, F1 = 0.74–0.75). As with classification of activity in Study 1, the ensemble methods provided the best performance for classifying user-reported mental focus. With limited data, the random forest classifier performed similarly to KNN (AUC = 0.85, F1 = 0.81). However, its performance improved when 95% of the data were used for training (AUC = 0.96, F1 = 0.90).

### 4.3. Discussion

In light of the challenge of controlling the level of a person’s focus on a specific activity in Study 1, we accompanied specific tasks with the participant’s self-reported measures of mental focus in this study. It is interesting that the strongest classifiers for user-reported mental focus (Study 2) were very similar to those for type of activity (Study 1). Specifically, the ensemble models were the best-performing of the black box models, with random forest providing the strongest classification performance (AUC = 0.85–0.96, F1 = 0.81–0.90). Although KNN was the best performing of the baseline models (AUC = 0.81–0.87, F1 = 0.79–0.80), we were surprised that a simple decision tree (depth = 4) also performed moderately well and was robust to limited data (AUC = 0.71–0.73, F1 = 0.74–0.75), which may suggest that this type of model would be useful in the early stages of training an individual’s EduFit device.

Logistic regression gives direct insight into the relative importance of the features by telling us how a standard deviation of change in one feature affects the outcome while holding the other features constant. The logistic regression model (Table 5) suggested that EDA was the most important feature (χ^2^(1) = 157.3, OR = 0.24) followed by TEMP (χ^2^(1) = 94.6, OR = 0.39). Although statistically significant (χ^2^(1) = 8.9, OR = 1.39), HR was of lesser importance. This yields insight into the general trends on how mental focus was manifested physiologically after controlling for individual differences. Given the large individual differences, these trends are not likely to provide a basis for strong classification. However, these general trends may be useful as a starting point, or informative prior, in the training of an individual’s device. The fact that decreased body temperature and EDA were associated with mental focus in our data suggests that concentration on a mental task is associated with a state of relaxation [6,30]. The association of mental focus with increased heart rate derived from the data suggests that these participants concurrently experienced a higher level of mental alertness as they were focused on the task [5]. Bringing this together, we can characterize user-reported focus on a task as a state of emotional and physical relaxation accompanied by mental alertness, which (simplistically) is expressed physiologically by decreased body temperature and EDA accompanied by an increase in heart rate.

## 5. Overall Discussion and Limitations

Just as physical fitness trackers have revolutionized the culture around physical health and exercise, the EduFit system may revolutionize studying by improving one’s awareness of unique study habits, including the level and duration of cognitive load used during study sessions. Although previous studies have shown that the concept of a fitness sensor to monitor studying is promising [5,6,7], no study has shown strong classification performance using the real-time measures that can be collected from ubiquitous wearable technology. We used the Empatica E4 similarly to [6,7], but the data suggest that any wearable technology that collects accurate and repeatable real-time measures for heart rate, EDA, and body temperature can be used to provide useful predictions for both the type of activity in which a student is engaged (Study 1) as well as whether or not the student is focused on a task (Study 2).

Automated feedback provided by fitness sensors has enabled people to track their exercise, lending a measure of accountability to their physical health. In the same way, when studying, it is important for a student to get feedback on whether or not she is actually engaged in a task and studying effectively. It is well-known that this metacognition is essential to effective personal development and learning [31], and real-time feedback provided by this type of wearable technology may help users develop the necessary personal awareness needed to study more effectively.

**Figure 1:**
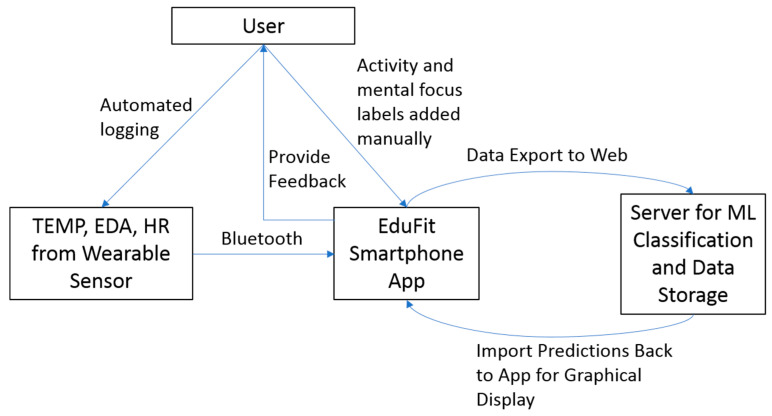
Conceptual diagram of the EduFit system, including the user, the wearable sensor, the smartphone app, and a web server.

Many aspects of implementation of this type of technology are straightforward. Similar to current fitness trackers, data from the wearable sensor can be exported to a server for processing, and then sent back to the user’s smartphone where graphics such as charts and tables representing the level of cognitive load and temporal trends can be presented to the user (Figure 1).

However, the models and relationships are less straightforward than with traditional fitness trackers. Models using accelerometer data to quantify magnitude and duration of movement, and those using heart rate to infer the aerobic activity of a person of specific gender and age, are relatively straightforward in comparison to quantifying cognitive load. Many confounding factors deteriorate these models, including the uniqueness of how we think and engage with different tasks, and react physiologically to our thought processes. To add further complexity, we have differences in how we interact with other aspects of our learning environments that are external to the task. Finally, these factors lead to errors in the training process itself. For example, our perceptions of whether or not we are focused will shift from person to person, and even from day to day. The extent to which this happens will affect any classifier trained with user-report data. In lieu of user-reports, observational data could be used to infer whether or not a person is focused. However, this would then contain observer error, which would likely be even greater than self-report error unless the observer is both expertly trained and knows the subject of observation well. Another option would be to go back to a design like Study 1 and thoughtfully link specific activities to specific mental demands. It may be straightforward to infer that completing a task like writing an essay or solving a math problem, which is aligned to a person’s ability level, would require mental focus. However, inferring that a task does not solicit mental focus is more challenging unless the participant self-reports it. For example, a person appearing to sit and do nothing may be daydreaming, or he may be thinking about a complex problem involving a higher level of cognitive load.

Although these questions of experimental design and causation pertaining to reliably training a system like EduFit will not go away anytime soon, we consider it promising that both Study 1, which relied on a person’s observed activity, and Study 2, which relied on self-reported mental focus, both demonstrated strong classification using KNN and the ensemble models, even in the case of limited training data. Given the limitations, we feel an effective way to start with this technology is to rely on user-reported data from the outset (Figure 1). We envision that in the beginning stages of implementation of EduFit with an individual, the user will control much of the labeling, including the types of activities of engagement as well as perceived level of cognitive load, thereby building a personalized workload monitoring system. These data can then be utilized to iteratively tweak the classification model so that it provides continually stronger predictions of the individual’s learning activities and cognitive load over time.

## Figures and Tables

**Table 1:** Summary of the three tasks used in Study 1, with cognition state and hypothesized level of cognitive load.

Cognition State	Cognitive Load	Associated Task
Deep Cognition	High	Playing Sudoku
Leisured Cognition	Moderate	Browsing the Internet
Daydreaming	Low	Sitting and Doing Nothing

**Table 2:** Descriptive statistics for each participant’s physiological measures across each activity in Study 1. N = number of measurements (1 measure per second), EDA = electrodermal activity, HR = heart rate, and TEMP = body temperature.

			EDA (μS)		HR (Beats/Minute)		TEMP (°C)	
Person	Activity	N	Mean	SD	Mean	SD	Mean	SD
1	Sudoku	1198	0.06	0.03	72.61	10.70	32.32	1.67
	Browsing Internet	1273	0.04	0.02	81.37	18.26	31.96	1.92
	Daydreaming	1359	0.05	0.01	68.55	7.68	31.16	1.53
2	Sudoku	9813	4.07	3.03	92.08	10.33	32.53	0.43
	Browsing Internet	9074	3.27	2.19	92.24	12.32	32.52	0.38
	Daydreaming	8860	2.81	2.68	88.74	12.90	32.55	0.33
3	Sudoku	4260	0.17	0.03	78.69	7.62	30.92	1.64
	Browsing Internet	3900	0.19	0.05	73.42	8.55	30.92	1.58
	Daydreaming	3229	0.19	0.08	73.50	8.15	31.56	0.60
4	Sudoku	2296	0.10	0.09	84.98	6.70	33.52	1.65
	Browsing Internet	2562	0.14	0.08	80.96	3.87	32.54	0.99
	Daydreaming	1735	0.09	0.01	89.97	3.85	34.49	0.78
5	Sudoku	2227	0.16	0.11	82.92	14.37	30.05	1.30
	Browsing Internet	2169	0.11	0.06	74.94	8.83	33.01	0.62
	Daydreaming	1804	0.05	0.04	71.89	11.35	31.93	2.53
6	Sudoku	2880	0.13	0.06	70.02	7.77	32.59	2.38
	Browsing Internet	3600	0.14	0.05	68.92	8.64	32.70	1.94
	Daydreaming	3420	0.14	0.08	74.29	7.60	32.06	2.61
7	Sudoku	10200	0.38	0.23	75.17	5.32	32.93	0.71
	Browsing Internet	3840	0.39	0.25	80.10	16.35	32.65	0.42
	Daydreaming	3540	0.43	0.44	73.85	6.25	32.31	0.89

**Table 3:** Classification performance of the test dataset. Models include the constant (outcome bias), baseline models (k-nearest neighbors (KNN), logistic regression, naïve Bayes, decision tree), and black box models (support vector machine (SVM), neural network, AdaBoost, and random forest).

Activity (Resting, Web, Sudoku)	Liberal (95% Training)				Conservative (10% Training)			
Model	AUC	F1	Precision	Recall	AUC	F1	Precision	Recall
Constant	0.49	0.22	0.16	0.40	0.50	0.22	0.16	0.39
K-Nearest Neighbors (k = 3)	0.97	0.93	0.93	0.93	0.89	0.84	0.84	0.84
Logistic Regression	0.52	0.23	0.44	0.41	0.36	0.28	0.41	0.41
Naïve Bayes	0.65	0.45	0.46	0.47	0.67	0.44	0.45	0.46
Decision Tree (depth = 4)	0.68	0.43	0.57	0.49	0.67	0.45	0.53	0.48
Support Vector Machine	0.50	0.31	0.33	0.32	0.44	0.28	0.31	0.29
Neural Network	0.79	0.61	0.61	0.61	0.33	0.45	0.47	0.47
AdaBoost	0.93	0.92	0.92	0.92	0.78	0.80	0.80	0.80
Random Forest	0.99	0.94	0.94	0.94	0.93	0.85	0.85	0.85

**Table 4:** Descriptive statistics for each participant’s physiological measures across the activities in Study 2. N = number of measurements (1 measure per second), EDA = electrodermal activity, HR = heart rate, and TEMP = body temperature.

			EDA (μS)		HR (Beats/Minute)		TEMP (°C)	
Person	Focused	N	Mean	SD	Mean	SD	Mean	SD
1	No	60	0.19	0.04	98.18	15.36	31.84	0.08
	Yes	514	0.22	0.02	74.74	7.05	32.29	0.13
2	No	0						
	Yes	855	0.09	0.03	97.94	18.28	33.68	0.43
3	No	117	0.07	0.01	80.29	4.37	30.85	0.06
	Yes	880	0.09	0.01	75.10	5.03	30.48	0.18
4	No	176	0.04	0.02	89.62	16.05	29.16	0.21
	Yes	583	0.05	0.02	90.47	8.59	30.69	1.44
5	No	0						
	Yes	475	0.20	0.07	79.36	9.74	32.33	0.15
6	No	293	0.38	0.08	71.40	10.63	33.53	0.35
	Yes	250	0.42	0.05	80.45	11.80	33.50	0.12
7	No	653	0.11	0.03	96.29	19.11	33.80	0.27
	Yes	238	0.06	0.02	101.41	13.67	33.38	0.58

**Table 5:** Binary logistic regression model for predicting a user’s reported state of focus based on EDA, body temperature, and heart rate. This model controls for variations in each participant’s intercepts and trends (χ^2^ = 4224.09, df = 31, *p* << 0.001).

Variable	B	SE	χ^2^ (df = 1)	*p*-Value	OR
EDA	−1.424	0.114	157.288	0.000	0.241
TEMP	−0.954	0.098	94.613	0.000	0.385
HR	0.332	0.111	8.941	0.003	1.394

**Table 6:** Classification performance of the test dataset. Models include the constant (outcome bias), baseline models (k-nearest neighbors (KNN), logistic regression, naïve Bayes, decision tree), and black box models (support vector machine (SVM), neural network, AdaBoost, and random forest).

Reported Focus (Yes/No)	Liberal (95% Training)				Conservative (10% Training)			
Model	AUC	F1	Precision	Recall	AUC	F1	Precision	Recall
Constant	0.46	0.64	0.56	0.75	0.50	0.64	0.56	0.75
K-Nearest Neighbors (k = 3)	0.87	0.80	0.80	0.80	0.81	0.79	0.79	0.79
Logistic Regression	0.54	0.64	0.56	0.75	0.52	0.64	0.69	0.75
Naïve Bayes	0.68	0.64	0.56	0.75	0.66	0.66	0.68	0.74
Decision Tree (depth = 4)	0.73	0.75	0.81	0.80	0.71	0.74	0.74	0.77
Support Vector Machine	0.51	0.61	0.61	0.61	0.53	0.64	0.62	0.67
Neural Network	0.54	0.64	0.56	0.75	0.52	0.64	0.68	0.75
AdaBoost	0.86	0.90	0.90	0.90	0.72	0.78	0.78	0.78
Random Forest	0.96	0.90	0.90	0.91	0.85	0.81	0.81	0.82
